# Distribution, functional impact, and origin mechanisms of copy number variation in the barley genome

**DOI:** 10.1186/gb-2013-14-6-r58

**Published:** 2013-06-12

**Authors:** María Muñoz-Amatriaín, Steven R Eichten, Thomas Wicker, Todd A Richmond, Martin Mascher, Burkhard Steuernagel, Uwe Scholz, Ruvini Ariyadasa, Manuel Spannagl, Thomas Nussbaumer, Klaus FX Mayer, Stefan Taudien, Matthias Platzer, Jeffrey A Jeddeloh, Nathan M Springer, Gary J Muehlbauer, Nils Stein

**Affiliations:** 1 Department of Agronomy and Plant Genetics, University of Minnesota, St. Paul, MN 55108, USA; 2 Department of Plant Biology, University of Minnesota, St. Paul, MN 55108, USA; 3 Institute of Plant Biology, University of Zurich, CH-8008 Zurich, Switzerland; 4 Roche NimbleGen, Inc, Madison, WI 53719, USA; 5 Leibniz Institute of Plant Genetics and Crop Plant Research (IPK), Gatersleben, D-06466, Germany; 6 Current address: The Sainsbury Laboratory, Norwich, NR47UH, UK; 7 Helmholtz Center Munich, German Research Centre for Environmental Health (GmbH), MIPS/IBIS, Institute for Bioinformatics and Systems Biology, 85764 Neuherberg, Germany; 8 Leibniz Institute for Age Research, Fritz Lipmann Institute, Jena D-07745, Germany

**Keywords:** Barley, Copy number variation, Comparative genomic hybridization, Disease-resistance genes, Double-strand break repair mechanisms

## Abstract

**Background:**

There is growing evidence for the prevalence of copy number variation (CNV) and its role in phenotypic variation in many eukaryotic species. Here we use array comparative genomic hybridization to explore the extent of this type of structural variation in domesticated barley cultivars and wild barleys.

**Results:**

A collection of 14 barley genotypes including eight cultivars and six wild barleys were used for comparative genomic hybridization. CNV affects 14.9% of all the sequences that were assessed. Higher levels of CNV diversity are present in the wild accessions relative to cultivated barley. CNVs are enriched near the ends of all chromosomes except 4H, which exhibits the lowest frequency of CNVs. CNV affects 9.5% of the coding sequences represented on the array and the genes affected by CNV are enriched for sequences annotated as disease-resistance proteins and protein kinases. Sequence-based comparisons of CNV between cultivars Barke and Morex provided evidence that DNA repair mechanisms of double-strand breaks via single-stranded annealing and synthesis-dependent strand annealing play an important role in the origin of CNV in barley.

**Conclusions:**

We present the first catalog of CNVs in a diploid *Triticeae *species, which opens the door for future genome diversity research in a tribe that comprises the economically important cereal species wheat, barley, and rye. Our findings constitute a valuable resource for the identification of CNV affecting genes of agronomic importance. We also identify potential mechanisms that can generate variation in copy number in plant genomes.

## Background

The identification and prevalence of copy number variation (CNV) among the genomes of individuals within a species has provided the rationale to redefine genomes as dynamic entities. Copy number variants (CNVs) are currently defined as unbalanced changes in the genome structure and include deletions, insertions, and duplications of >50 bp in size [1].

The first studies documenting the existence of numerous CNVs throughout the human genome and their relation with genetic disorders [2,3] were followed shortly by the completion of the first CNV map of the human genome [4]. Since then, an increasing number of human studies have produced evidence for the association of CNV with complex diseases, environmental response, and population diversity (reviewed in [1]). Other large-scale studies showed that CNV is common in other animal genomes including chimpanzee and other great apes [5,6], cattle [7,8], rat [9], dog [10,11], and *Drosophila *[12] among others.

CNV is also a common feature of plant genomes and several recent studies provided insights into the extent of this type of intraspecific structural variation in plants. High levels of CNV have been found distributed throughout the maize genome, with a tendency for variants to be located near the ends of the chromosomes and the existence of high- and low-diversity regions [13-15]. The undomesticated progenitor of maize (teosinte) exhibits high levels of CNV and shares most of the variants with modern maize [15]. There is evidence that prevalent CNV in maize plays an important role in contributing to phenotypic variation as it overlaps loci associated with important traits related to stress and stimulus responses [16]. Studies in other plant species including Arabidopsis [17,18], wheat [19], sorghum [20], rice [21,22], and soybean [23,24], also demonstrated that CNV contributes to the genetic diversity of their genomes. Genes affected by CNV in soybean are enriched for annotations related to stress and plant defense responses [24]. There are several examples demonstrating a causal relationship between CNV and plant phenotypes. CNV at the *Rhg1 *locus in soybean increases the resistance to the cyst nematode *Heterodera glycines *[25]. In barley, increased copy number at the boron transporter gene (*Bot1*) confers boron-toxicity tolerance to the African barley landrace 'Sahara' [26]. CNV at the *MATE1 *transporter gene in maize is associated with increased aluminum tolerance [27].

CNV can arise from a variety of molecular mechanisms including: non-allelic homologous recombination (NAHR) at regions of extensive sequence similarity (synonymous with unequal crossing-over); non-homologous end-joining (NHEJ) and microhomology-mediated end-joining (MMEJ), which are associated with DNA repair at regions with very limited or no homology; replication-error mechanisms such as fork stalling and template switching (FoSTeS) and microhomology-mediated break-induced replication (MMBIR); and transposable element (TE)-mediated mechanisms [28-31]. CNV could also arise from the segregation of non-allelic homologs (SNH) among F2 siblings or recombinant inbred lines (RILs) [32,33]. NAHR is one of the best studied recombination-based mechanisms in humans, known to cause recurrent rearrangements in hotspots of homologous recombination, while replication mechanisms are a major contributor to non-recurrent CNVs [31]. In contrast, our understanding of the most prevalent contributors to CNV in plants is more limited.

Barley (*Hordeum vulgare *L.) is one of the first crops domesticated by humans approximately 10,000 years ago [34] and currently ranks fourth among cereals in terms of harvested area [35]. It is also considered a model for the *Triticeae *tribe, which includes other agronomically-important species such as wheat and rye. CNV is known to affect some genes with important adaptive functions in barley. As mentioned above, increased copy number of a boron transporter gene (*Bot1*) confers boron-toxicity tolerance [26]. *CBF *(*C-Repeat Binding Factor*) gene copy number variation at the *Frost Resistant-2 locus *(*FR-2*) is associated with low-temperature tolerance [36]. These examples, together with the recent discovery of CNV affecting two major genes controlling flowering time in wheat, *Ppd-B1 *and *Vrn-A1 *[37], suggest CNV as a potential source of agronomically important phenotypic variation in barley and other *Triticeae *crops.

In the present study, we developed and used a barley comparative genomic hybridization (CGH) array containing 2.1 M probes covering approximately 50 Mbp of repeat-masked barley sequence (cv. Morex). Fourteen barley genotypes including cultivars (*H. vulgare *ssp. *vulgare*) and wild barleys (*H. vulgare *ssp. *spontaneum*) were compared to the 'reference' genome of cv. Morex [38] to survey the landscape of CNV in the barley genome. The wild barley accessions allowed us to evaluate the impact of domestication and selection on the extent of overall CNV in the genome. The availability of additional sequence data from one of the cultivars surveyed by the CGH array (cv. Barke) permitted further exploration of the structural variants at the nucleotide level and provided insights into the mechanisms contributing to CNV in barley. The CNVs discovered in this study represent the first catalogue of this type of structural variation in barley to date, which provides the opportunity to characterize the types of genes affected by CNV and opens the door for future research on this type of genomic diversity in barley and other highly syntenic genomes such as rye or wheat.

## Results

### Development and validation of the barley CGH array

Comparative genomic hybridization (CGH) provides a robust method for detecting CNVs [39]. We developed a high-density oligonucleotide microarray containing 2.1 million probes derived from low-copy sequences in 115,003 whole-genome shotgun (WGS) contigs of the barley reference genome Morex (see Materials and Methods). The array design selected 200 bp regions that were separated by at least 500 bp (visualization of array design provided in Additional file 1, Figure S1). For each 200 bp fragment (thereafter called 'contig fragment') the array included 10 long oligonucleotide probes of 56- to 100-mers (median length of 76 bp). This design strategy allowed for reliable detection of relatively small CNVs and coverage of the low-copy regions of the genome. The barley CGH custom array included probes for 211,669 200 bp contig fragments on 115,003 WGS contigs. Most of these 115,003 contigs (60.2%) were represented by one fragment, 19.7% by two fragments, and the remaining 20.1% of the contigs were represented by three to 19 fragments of 200 bp (Additional file 2, Table S1). The contig fragments from the same WGS contig are generally separated by 500 bp unless there are repetitive sequences and then the spacing between adjacent fragments can be longer. The actual distance between fragments on different WGS contigs cannot be calculated as the distance between contigs is not known. The array includes probes for all types of low-copy sequences and the ratio of exon and non-exon probes is 1:3.2.

The recently released barley physical map [38] was used to assign chromosomal positions to the contig fragments surveyed by the array. A total of 88.7% of the contig fragments could be assigned to chromosome 1H-7H bins, and 33.7% could also be assigned to a specific genomic location.

To test the utility of the barley CGH array for detecting specific regions of the barley genome we conducted an experiment with the cv. Betzes and a wheat-barley chromosome addition line (CS-3HL), which carries the barley 3HL chromosome arm of cv. Betzes in the genetic background of wheat cv. Chinese Spring (CS-3HL) [40]. Equal amounts of Betzes and CS-3HL DNAs were hybridized to arrays to check if the additional genomic content corresponding to 3HL could be detected by the CGH array. Chinese Spring (CS) wheat and Betzes barley were hybridized to the array as a control. The log2 (CS-3HL/Betzes) signal intensities of all contig fragments on the array were displayed by chromosome/chromosome arm and the expected increased hybridization signals for chromosome 3HL were observed (Additional file 1, Figure S2A). In contrast, CGH comparison of CS and Betzes did not reveal any chromosomal regions with biased signal (Additional file 1, Figure S2B).

### Identification and distribution of CNV

To detect CNV among barley genotypes, we performed CGH on 14 barley accessions relative to the reference genotype Morex. The 14 accessions were chosen to represent barley diversity and included eight barley cultivars (*H. vulgare *ssp. *vulgare*) and six wild barleys (*H. vulgare *ssp. *spontaneum*, progenitor of cultivated barley) (see Materials and Methods, Additional file 1, Figure S3 and Additional file 2, Table S2 for more information about the accessions used). Following normalization of the hybridization signals, the average ratio (log2) of each sample relative to Morex was calculated for the 211,669 200 bp contig fragments that were each represented by 10 probes. By testing these 10-probe regions of 200 bp as a group, it was possible to reduce the influence of small sequence polymorphisms on the identification of structural variation. The 200 bp regions that exhibit CNV were identified using the expectation maximization (EM) algorithm followed by the application of minimum change in log2 ratio (± 0.9) that requires a near two-fold change in signal intensity. Events were then classified based on whether they exhibited higher signal than Morex (UpCNV) or lower signal than Morex (DownCNV/PAV) (Additional file 2, Table S3). We grouped together DownCNV and presence/absence variation (PAV) because the array cannot distinguish between these types, as a lower intensity signal in another genotype relative to Morex is observed in both cases. PCR-based validation for 148 DownCNV/PAV events suggested that 77.7% of these (115 events) may actually represent PAVs (See 'Validation of structural variants' and Additional file 2, Table S4 for more information). It is worth noting that the design of a microarray based on a single reference genome often results in biased detection of more DownCNV/PAV than UpCNV. This is due to the fact that all sequences on the array must be represented in the reference genome but some of these may be missing from other genotypes. The sequences that are present in other genotypes but missing from the reference genome are not surveyed in this type of experiment.

**Figure 1:**
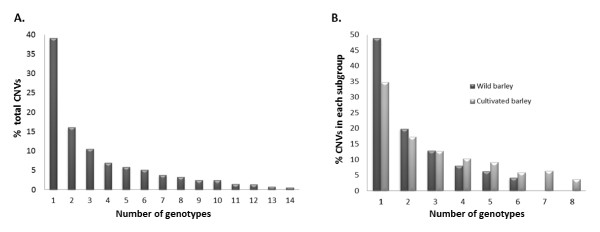
**Frequency spectrum of CNV**. (**A**) Percentage of CNVs identified in one to 14 genotypes relative to the total number of events; (**B**) frequency spectra comparison between wild and cultivated barley.

The application of the criteria described above identified 31,494 contig fragments (14.9% of all tested regions) that are affected by structural variation in at least one genotype relative to Morex (Table 1; Additional file 2, Table S3). In the wild accessions, approximately 4.5% of the regions tested exhibit structural variation, while the proportion of regions with structural variation was lower and more variable in the domesticated barleys (Table 1). The frequency spectrum of CNV reveals that 39.1% of the variants identified were present in only one of the tested genotypes (singletons) while the remaining 60.9% were found in two or more genotypes, with 181 variants (0.6%) present in all 14 genotypes relative to Morex (Figure 1A). Most of those 181 variants (91.2%) were DownCNV/PAVs which, most likely, represent unique sequences in the reference genome 'Morex'.

**Table 1:** Number and percentage of copy number variants for each genotype compared to Morex.

Contrast	UpCNV	DownCNV/PAV	Total	Events with CNV (%)
Barke	612	6,081	6,693	3.2
Betzes	606	5,620	6,226	2.9
Harrington	464	5,767	6,231	2.9
Haruna Nijo	500	6,000	6,500	3.1
Bowman	440	4,655	5,095	2.4
Igri	462	6,874	7,336	3.5
Steptoe	449	6,335	6,784	3.2
Franka	506	5,857	6,363	3.0

Total cultivated barley	_^a^	_^a^	16,918	8.0

Hsp11	827	8,487	9,314	4.4
Hsp248	821	8,709	9,530	4.5
Hsp278	768	8,470	9,238	4.4
Hsp357	875	8,666	9,541	4.5
Hsp462	861	8,759	9,620	4.5
Hsp730	834	8,384	9,218	4.4

Total wild barley	_^a^	_^a^	26,200	12.4

All genotypes	_^a^	_^a^	31,494	14.9

The total numbers and percentages of CNVs considering all cultivated barleys, wild barleys, and genotypes are also shown.

^a^Numbers not displayed as one contig fragment can be an UpCNV in one of the genotypes of the category and DownCNV/PAV in another.

**Figure 2:**
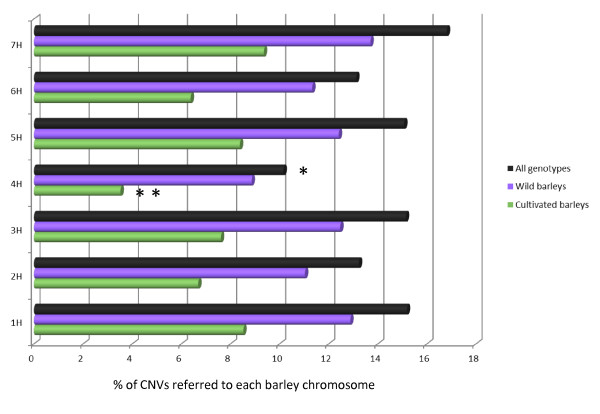
**Distribution of CNV per chromosome for all genotypes, wild barleys, and cultivated barleys**. The bars represent percentages of CNVs assigned to each chromosome relative to the total number of contig fragments present on the corresponding chromosome. The single asterisk indicates that, considering all genotypes, the percentage of CNV on 4H is significantly lower compared to other chromosomes (t-test *P *value = 0.0002), while the double asterisk indicates the frequency of CNV on 4H in cultivated barley is significantly lower than wild barley (*P *value = 0.003 by t-test).

The chromosomal distribution of CNVs was assessed by calculating the percentage of 200 bp regions mapped to each chromosome that exhibit structural variation (Figure 2; Additional file 1, Figure S4). Since barley chromosomes have different lengths and are represented on the array by different numbers of contig fragments, percentages of CNVs refer to the total number of sequences tested on each chromosome. As Figure 2 shows, the percentage of CNV on chromosome 4H is significantly lower than for all other chromosomes (t-test *P *value = 0.0002) and most of those variants were rare (52.4%), while chromosomes 1H and 7H contained the highest frequency of CNVs (Figure 2).

**Figure 3:**
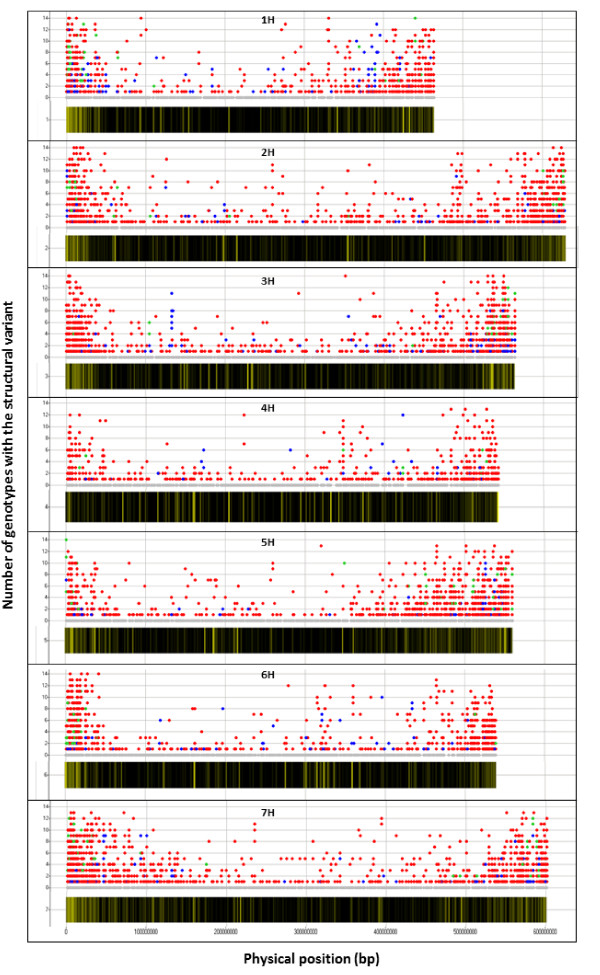
**Distribution and frequency of structural variation across the seven barley chromosomes**. The upper plots show, for each barley chromosome, all variants assigned to chromosome positions and the number of genotypes sharing each variant, with colors indicating the type of structural variation (blue=UpCNV; red=DownCNV/PAV; green=Up and Down; grey=no variation). The lower panels shown for each chromosome illustrate the proportions of copy number variants per 1.5M bp window with respect to the total number of fragments assigned to that window, with proportions represented by a color gradient from black (proportion =0) to yellow (proportion = 1).

**Figure 4:**
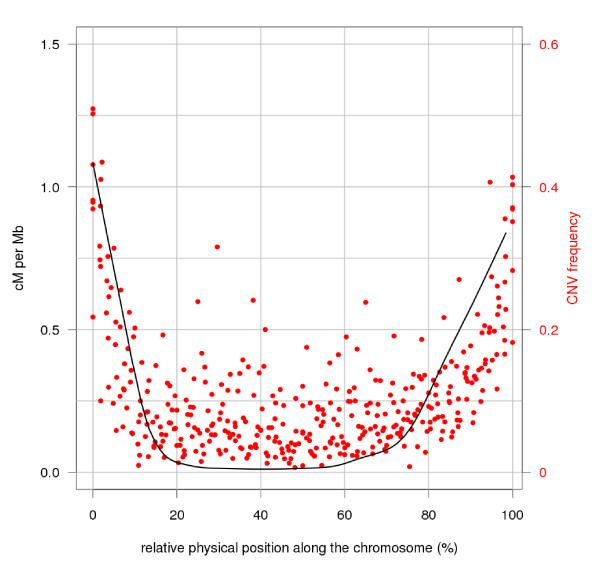
**Relationship between recombination rate and frequency of CNV**. The black line represents the recombination trend calculated from the cM/Mb ratios along the physical map. All the chromosomes were combined and the window size was set to 10 Mb. The red dots represent the proportion of CNVs with respect to the total number of contig fragments in each 10 Mb bin.

The analysis of the physical position of structural variants reveals more variants towards the ends of all seven chromosomes (Figure 3, upper plots; Additional file 1, Figure S5). The telomeric regions also have a higher density of shared variants (Figure 3, upper plots). This could be a function of the increased number of contig fragments near the ends of the chromosomes. However, an analysis of the frequency of structural variants in 1.5 Mbp sliding windows (Figure 3, lower plots) revealed that the proportion of variants was higher towards the ends of all chromosomes but 4H. On chromosome 4H, a more even distribution of the CNVs is observed. A comparison of the genetic and physical map [38] showed a moderate correlation (Spearman's rho = 0.54) between recombination rate and frequency of CNVs (Figure 4). Our analysis also identified several regions identical by descent that completely lack CNV. For example, cv. Bowman has a complete absence of CNV on the distal end of 7HS (Additional file 1, Figure S5) and this cultivar is related to Morex by pedigree. A lack of single-nucleotide variation (SNV) in the same region was also observed by survey sequencing [38].

The lack of a fully assembled genome sequence reduced our ability to assess the exact size for many of the CNVs. However, there were many examples in which multiple adjacent 200 bp regions on the same WGS contig showed similar CNV patterns. There are 7,732 CNV events in which at least two consecutive contig fragments exhibit similar patterns. Hundreds of these variants are composed of three to eight adjacent regions. An example of four DownCNV/PAVs covering a 4.4 kb region of chromosome 2H is shown in Additional file 1, Figure S6.

### Validation of structural variants

Spatial bias in DNA microarray hybridizations is still a general problem that can affect the results [41]. To test if the position of the probes on the array substantially affected their intensity signals and to confirm our estimates of CNV, we designed a second array that contained the same probes in a different layout. Seven of the same genotypes (Barke, Betzes, Bowman, Haruna Nijo, Steptoe, Hsp11, and Hsp 730) were hybridized to this array. The percentage of common CNVs that were identified in this independent analysis was quite high for each genotype: 93.8% for Barke, 98.3% for Betzes, 99.7% for Bowman, 96.9% for Haruna Nijo, 95.8% for Steptoe, 97.5% for Hsp11, and 98.5% for Hsp730, indicating that spatial bias did not significantly impact our results and providing validation for the CNV that were discovered.

Semi-quantitative PCR assays for 26 contig fragments showing DownCNV/PAVs in at least one genotype (148 total DownCNV/PAVs), and qPCR assays for 17 contig fragments affected by UpCNV (55 total UpCNVs) were conducted to validate the CGH array results. The majority (25/26) of DownCNV/PAV events were validated in the majority of genotypes (18 matched CGH data in all 14 genotypes, six regions were validated in 13 genotypes, and one region was validated in 12 genotypes). Only one of the contig fragments affected by DownCNV/PAV could not be validated by semi-quantitative PCR (Additional file 1, Figure S7A; Additional file 2, Table S4). Based on PCR results, most of these variants were presence/absences (77.7%) (Additional file 1, Figure S7A; Additional file 2, Table S4). From the 17 UpCNV contig fragments surveyed by qPCR, seven exhibited total correspondence to CGH data in all tested genotypes and almost all the remaining regions could be validated in >10 genotypes (Additional file 1, Figure S7B; Additional file 2, Table S4).

### Functional impact of CNV

Contig fragments on the array were annotated relative to predicted barley genes [38]. We found 58,791 contig fragments (27.8% of the array) with at least one gene prediction, and 39,574 of those were matching transcriptionally active high-confidence (HC) genes [38]. Functional annotations and gene ontology (GO) terms for the three main categories 'biological process' (BP), 'cellular component' (CC), and 'molecular function' (MF) were obtained for the HC genes on the array (Additional file 2, Table S3).

The high level of CNV among barley genotypes has the potential to influence phenotypes through changing gene dosage. A comparison of the CNVs relative to annotated genes identified a total of 5,629 CNVs affecting exons (9.5% of the exon sequences on the array). There were 2,194 CNVs that affected 1,585 genes that are highly conserved across grass genomes (9.0% of HC genes on the array) (Additional file 2, Table S3). We assessed the frequency of exons in the contig fragments affected by UpCNVs and in those affected by DownCNV/PAV. Noteworthy, the proportion of UpCNVs that affected coding sequences (30% of all UpCNVs) was higher than the proportion of DownCNV/PAV (16.4% of all DownCNV/PAVs). This higher relative representation of exons within the UpCNVs identified could reflect the fact that many of the sequences assayed are single copy and therefore a DownCNV/PAV would result in the lack of an essential gene product, which may have deleterious consequences. In contrast, these coding sequences may tolerate duplication in some genotypes relative to Morex.

GO-term enrichment analysis revealed that genes affected by CNV are enriched for genes belonging to categories 'cell death' and 'protein modification'. The majority of the 'cell death' genes were disease resistance (R) genes encoding nucleotide-binding site leucine-rich repeat (NBS-LRR) proteins, the most abundant class of R-proteins which are involved in pathogen recognition and signaling initiation [42,43]. Although protein kinases, which mediate most of the signal transduction in eukaryotic cells, were predominant in the category 'protein modification', other classes of R genes encoding Ser/Thr kinases, receptor-like kinases (RLKs), and receptor-like proteins (RLPs) are also included in this category. The chromosome location of the CNVs overlapping R genes indicated the tendency of these gene families to be clustered in the genome, with the distal ends of 1HS and 7HS containing the highest number of variants (Additional file 1, Figure S8).

### CNV between and within wild and cultivated barley

**Figure 5:**
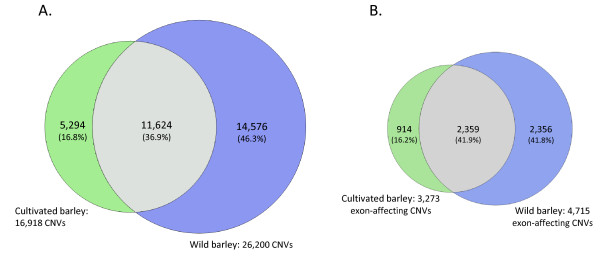
**Comparison between CNVs identified in wild and cultivated barley**. (**A**) Venn diagram showing the overlap between regions affected by CNV in both subgroups. (**B**) Venn diagram illustrating the overlap in CNVs that affect coding sequences.

A total of 16,918 CNVs (8% of the regions represented on the array) were identified in cultivated barley (*H. vulgare *ssp. *vulgare*), and 26,200 variants (12.4% of regions) were identified in its wild ancestor *H. vulgare *ssp. *spontaneum *(Table 1). Almost half of the CNVs found in the study were present only in wild barley (14,576 variants; 46.3%), while just 16.8% of the events (5,294 CNVs) were exclusive of cultivated barley (Figure 5A). The remaining 36.9% of the variants (11,624) were present in both wild and cultivated barley. We also calculated the frequency spectrum of CNV within each subspecies (Figure 1B). Both spectra were very similar, although wild barley had higher percentages of unique structural variants than cultivated barley (48.8% *vs*. 34.8%), which could be a consequence of the lower number of wild barleys considered in the study. Percentages of those 'rare' events were fairly evenly distributed among the wild barley accessions and ranged between 12.6% (Hsp278) and 18.5% (Hsp11). However, the numbers of unique variants in the 'cultivated barley' subgroup were more variable, with Steptoe contributing 28% of the unique events in domesticated barley, followed by Igri (16.2%), Haruna Nijo (14.7%), Barke (14.6%), and Franka (12.3%). Betzes, Bowman, and Harrington exhibited the lowest percentage (approximately 4%) of unique events.

As Figure 2 shows, all chromosomes had lower levels of CNV among cultivated varieties, although it was more pronounced in chromosome 4H (t-test *P *value = 0.003). Noteworthy, almost all the 'rare' variants located on 4H were found only in wild barley. To test for possible differences in the pattern of CNV between both subspecies, we calculated the difference between the number of variants present in wild and cultivated barley along 1.5 Mbp windows, and the difference was displayed for all seven barley chromosomes (Additional file 1, Figure S9). As expected, positive value peaks were more frequent and more widely distributed than the negative values indicating that, in general, wild barley has higher CNV diversity throughout the genome. However, distal chromosomal regions showed more pronounced differences between domesticated and undomesticated barley. The most extreme example is the region on the long arm of 5H where the CNV reduction in cultivated barley was more prominent and extended longer (Additional file 1, Figure S9). This chromosome has undergone intensive selection since it contains many important domestication-related traits such as dormancy and flowering time, as well as malting quality traits that modern breeders have selected [44].

A comparison between CNVs overlapping exons in wild and domesticated barley revealed that >80% of the variants affecting genes were present in wild barley (4,715 CNVs) and half of those were also found in cultivars (Figure 5B). Only 914 exon-affecting CNVs (16.2%) were found only in cultivars. These percentages are similar to those found in the total CNV comparison (Figure 5A), suggesting that, although a tendency for retaining coding sequences is observed, the reduction of CNV diversity was not markedly favoring coding or non-coding sequences.

### Confirmation of CNV at the sequence level

The availability of a high-quality WGS assembly from cv. Barke provided an opportunity to study the CNVs identified in this cultivar relative to Morex at the DNA sequence level. To perform a rigorous analysis of the specific sequence changes in the detected CNVs, we selected those WGS contigs that were represented by multiple contig fragments for which a Barke-Morex UpCNV or downCNV/PAV affected the internal fragments but did not affect flanking fragments. A total of 409 Morex WGS contigs containing 703 DownCNV/PAVs, and 42 Morex WGS contigs containing 69 UpCNVs met those criteria and were subsequently aligned to the cv. Barke WGS assemblies. The closest homolog(s) in the Barke WGS assembly was identified for each of the selected Morex contigs. It should be noted that, due to the more fragmented nature of the Barke assembly, a single Morex contig usually corresponded to multiple Barke contigs.

**Figure 6:**
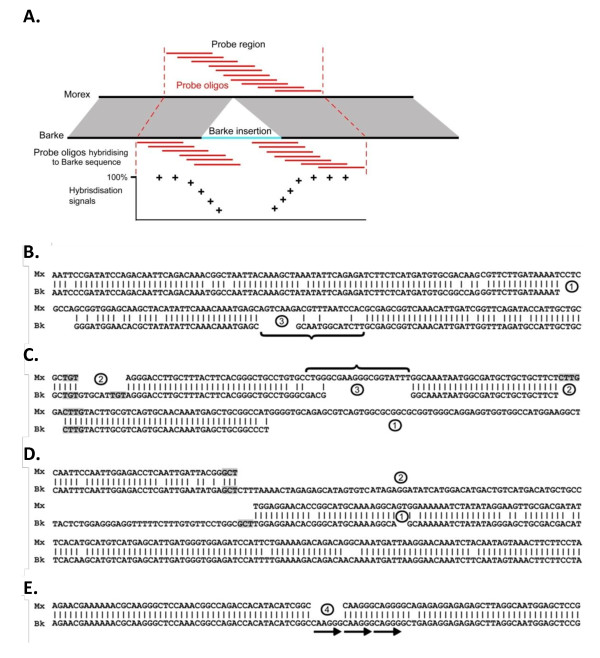
**Examples of sequence alignments of contig fragments containing DownCNV/PAVs**. The sequence of the barley cultivar Morex is shown at the top and the sequence of cultivar Barke at the bottom. (**A**) Schematic representation of how an insertion in Barke can lead to a DownCNV/PAV call. Sequence regions that are orthologous are connected by shaded areas. The additional sequence in Barke is depicted in light blue. The full contig fragment is composed of 10 overlapping probes. Those probes which overlap the breakpoint of the insertion will produce a low intensity signals or no signals, resulting in a reduced overall signal of the targeted contig fragment. (**B**) Contig fragments with multiple insertions/deletions. (**C**) Contig fragment with multiple deletions, including one that expands past the border of the fragment. (**D**) Contig fragment that contains additional sequences in Barke. (**E**) Contig fragment that contains an insertion/deletion that most likely originates from template slippage. The numbers in circles identify different types of insertions/deletions: 1, insertion/deletion that contains no obvious signature; 2, insertion/deletion that shows a typical signature of double-strand break repair via single-strand annealing (SSA); 3, insertion/deletion which contains filler sequence (indicated by a curly bracket) and that presumably is the result of DSB repair via synthesis-dependent strand annealing (SDSA); 4, insertion/deletion originated from template slippage of direct repeats (indicated by arrows).

In a total of 337 of the 703 downCNV/PAV regions, we were able to identify putative orthologous sequences in cv. Barke that could be aligned across the entire region of the CNV (that is, the CNV region was completely covered by the cv. Barke assembly, allowing for detailed analysis of the CNV borders, see below). It is worth noting that DownCNV/PAVs are likely to cause difficulties in performing high-quality alignments and the low rate of finding orthologous sequences from cv. Barke may result from DownCNV/PAV. The majority (76%) of the DownCNV/PAVs were supported by the sequence alignments (Table 2). In 114 cases, the contig fragment was completely absent, while flanking regions were still present. In 143 cases, the contig fragment was at least partially absent (Table 2). In 80 cases (24%), the entire contig fragment was present at a sequence identity of at least 95% and without insertions/deletions >1 bp and was considered false positive. Interestingly, 10 of these 80 contig fragments contained insertions in Barke, which ranged from 22 to 218 bp in size. These results indicate that, in some cases, the presence of an insertion can lead to DownCNV/PAV signals in CGH experiments (see Discussion and Figure 6A).

**Table 2:** Analysis of Morex and Barke sequence alignments in regions showing CNV

Sequence present in Barke (%)	Fragments with NoCNV (*n*, %)	DownCNV/PAVs (*n*, %)	UpCNVs (*n*, %)
0	156 (6)	114 (34)	7 (11)
0-24	31 (1)	16 (5)	0 (0)
25-49	42 (2)	18 (5)	3 (5)
50-74	59 (2)	25 (7)	2 (3)
75-99	280 (10)	84 (25)	8 (13)
100	2,130 (79)	80 (24)	49(79)

Total number of contig fragments analyzed	2,698 (100)	337 (100)	69 (100)

For all contig fragments showing UpCNV and DownCNV/PAV, we calculated the percentage of the sequence that was present in Barke. Contig fragments not affected by CNV (NoCNV) were also analyzed.

The alignment analysis of the 69 UpCNVs showed that in 49 cases (71%) the contig fragment was entirely present in Barke, while in 29% the contig fragment was partially absent. We did not further investigate whether multiple copies of the contig fragments were present in Barke due to the high level of difficulty in assigning the Morex reference sequence to one particular Barke copy in an automated manner. In addition, we analyzed 2,698 contig fragments that had no structural variation. We found that the vast majority (79%) of those fragments were present in full length in Barke (Table 2). These results indicate that the number of false negatives is relatively low.

### Molecular mechanisms of CNV formation

The alignments of the Morex and Barke sequences for the DownCNV/PAVs which contained insertions/deletions were analyzed to further study the molecular mechanisms that produced CNV. In total, we identified 299 insertions/deletions in the 200 bp contig fragments targeted by the array probes. Some of those affected only parts of the contig fragment while, in others, the entire fragment plus the flanking regions were absent. The insertions/deletions ranged from 1 bp to >7 kb, with an average of 492 bp. Most insertions/deletions were relatively short, as 162 of them (54%) were <100 bp.

The sequences of the insertions/deletions and their flanking regions were analyzed to obtain indications of their mechanistic basis (examples in Figure 6). There are sequence signatures suggesting double-strand break (DSB) repair via single-strand annealing (SSA) in 123 cases (41.1%). These are short sequence motifs of 2 to 40 bp which are exactly bordering the breakpoint of the deletion and are repeated at the other end inside the deleted region (Figure 6C and 6D). A comparison with simulations of randomly generated sequence insertions/deletions provides evidence that the signatures >2 bp are highly significantly over-represented in the dataset analyzed, while those with no or 1 and 2 bp signatures were strongly under-represented (*P *<0.0001). This indicates that the observed sequence signatures are indeed the products of DSB repair and do not appear by mere chance.

Segments of non-homologous sequences are present in 38 insertion/deletions (12.7%). This means that in the region of the insertion/deletion, the sequences of the two cultivars are completely different from each other and cannot be aligned (Figure 6B and 6C). These non-homologous stretches were likely introduced during DSB repair via synthesis-dependent strand annealing (SDSA) when copies of non-homologous sequences are used to repair a DSB. There are 47 insertions/deletions (15.7%) ranging from 1 to 6 bp that are attributed to template slippage. That is, the complete sequence of the insertion/deletion is repeated perfectly in the immediate flanking region (example in Figure 6E). There were 91 insertion/deletions (30.4%) for which no mechanism could be ascribed.

## Discussion

We report on the first comprehensive study on CNV in the genome of barley, a diploid *Triticeae *species. WGS sequences from the barley reference genotype 'Morex' [38] were used to design a CGH long-oligonucleotide array that covered 50 Mbp of repeat-masked barley genome sequence that was capable of detecting CNVs as small as 200 bp with a very high confidence. This type of array design has proven to be successful in discovering structural variants in the genome of other species (for example, [5,11,15]) and it has also been used for the characterization of mutants [45] and for high-throughput genotyping in complex genomes [46].

Here, we have surveyed the landscape of CNV in a representative panel of both cultivated and wild genotypes to discover commonalities and differences between modern barley and its undomesticated progenitor regarding this type of genomic structural variation. The use of two array designs supported the reproducibility of the results obtained. A combination of PCR assays and sequence analyses validated the majority of the detected variants that we tested. Also, as found in other studies (for example, [47]), frequency spectrum of CNV resembles that of other genetic variants such as SNPs, where most variants are at low frequency. Spectra of CNVs are similar for wild and cultivated barleys and corroborate the quality of our dataset.

### High levels of CNVs in the barley genome are located preferentially in regions of high recombination

Our diverse panel of fourteen genotypes detected 31,494 CNVs representing 14.9% of the barley contig fragments that were surveyed. This is a high percentage, over the 10% found by similar CGH testing of the maize genome [15], one of the most diverse crops. However, the maize study used a gene-based CGH design, while our custom array also included non-coding regions. If only annotated contig fragments are considered, the percentage of CNV affecting genes decreases to 9.5%, which is similar to observations in maize. Although comparison with other species and/or studies is more complicated given the differences in experimental designs and analyses, the number of CNVs identified is high considering that barley is a diploid species with a very low outcrossing rate (0% to 1.8%; [48]). Although our array design prioritizes the detection of small structural variants, analysis of contigs containing many targeted fragments revealed that only 39.7% of the variants are >200 bp. This is in agreement with our observations from survey sequencing of cultivars Morex and Barke, where more than half of the identified insertions/deletions were <100 bp. Similarly, Swanson-Wagner *et al. *[15] found most structural variants affecting single genes in maize.

The recent availability of a physical map of the barley genome allowed the assignment of most of the CNVs to physical positions and/or chromosomes [38], which enabled us to explore the genomic distribution of the CNVs identified. In general, CNVs were much more frequent at the end of all barley chromosomes, which we found mirrored the meiotic recombination rate. A previous analysis of single nucleotide variation (SNV) in barley also showed a similar pattern [38]. This correlation between SNV and CNV frequency has been previously observed in other studies [24]. Barley chromosome 4H is a special case, with both significantly lower SNV and CNV frequency. Furthermore, the proportion of CNVs on this chromosome is not higher towards the ends of the chromosome arms. Since recombination-based mechanisms such as NAHR are a main cause of recurrent rearrangements [6,11,29,31], the reduced meiotic recombination rate on chromosome 4H and on centromeric and peri-centromeric regions of all chromosomes [38] can limit the emergence of structural variants. Similarly, this reduced recombination frequency can reduce CNV diversity by extending the effect of the background selection against deleterious variants [49].

### Depletion of CNV diversity during barley domestication and breedingb

Barley was domesticated approximately 10,000 years ago from its wild progenitor *Hordeum vulgare *ssp. *spontaneum *and, since then, has been subjected to extensive selection and breeding, which has severely reduced SNV diversity [50,51]. The use of six wild barley accessions in this study allowed us to evaluate the impact of domestication and breeding practices on CNV diversity. Unlike maize, where high percentages of shared CNVs between domesticated and undomesticated accessions were reported [15], we found that almost half of the CNVs identified are present only in the wild ancestor of cultivated barley. Although the fact that the barley CGH array is based on a barley cultivar (cv. Morex) may favor the detection of PAVs in genotypes that are distantly related to the reference, we also find high numbers of UpCNVs (which are not affected by this bias) in wild barley accessions (Table 1). The use of a domesticated barley accession sequence for the array design limits our capability to detect wild barley sequences that are not present in cultivated barley. Therefore, we are likely underestimating the number of CNVs present in wild barleys.

Our findings support the loss of genetic diversity as a consequence of barley domestication and extensive breeding and indicate that those bottlenecks also affect CNV diversity. Chromosome 4H suffered the biggest reduction in CNV diversity, which may be related to its reduced effective recombination rate (see above). The presence of both exonic and non-exonic sequences on the custom array allowed us to investigate if the reduction in CNV diversity was preferably occurring in the non-coding regions of the genome. We found no tendency to retain exons as percentages of unique and shared CNVs and exon-affecting CNVs in wild and cultivated barley were comparable.

### CNV can be the result of DNA repair and template slippage

Although recent CNV surveys in plants are increasing our knowledge of the extent and patterns of CNV in plant genomes (for example, [15-17,20,24]), we have a limited understanding of the most prevalent mechanisms for CNV formation in plants. A sequence based comparison of Barke-Morex CNVs showed that, in >41% of the deletions analyzed, diagnostic sequence signatures of double-strand breaks (DSBs) repaired via single-stranded annealing (SSA) were found. These signatures, which were previously attributed to 'illegitimate recombination', have been found in maize flanking the short deletions (5 bp to 178 bp) occurring during the process of fractionation [52]. These authors observed that, as previously noted in a tetraploid Arabidopsis ancestor [53], these deletions removed preferentially genes from one of the two homeologs to eliminate genetic redundancy. Our study shows that this short deletion mechanism is also frequently occurring in a diploid species such as barley. The Barke - Morex sequence comparisons also found evidence that 13% of deletions contained 'filler' segments which point to a DSB repair via synthesis-dependent strand annealing (SDSA; [54]). Previous studies showed that DSB repair is a frequent cause of sequence variation in plants [55,56]. However, the present dataset allowed for the first time the frequency of such events to be quantified. Furthermore, we identified template slippage as a candidate mechanism for almost 16% of the deletions analyzed.

The lack of WGS assemblies of sufficient quality and length from other genotypes, especially from wild barley accessions, did not allow for a robust sequence comparison as the one performed with Barke. However, partial sampling of a WGS assembly of the barley cultivar Bowman revealed similar results, indicating the same molecular mechanisms (data not shown).

Although the barley CGH array did not allow us to explore genomic regions of extensive sequence similarity, other processes such as NAHR can contribute to barley CNV formation. Similarly, TE insertions could cause CNV. Although we carefully chose unique probes for the array design to avoid TE-related sequences, some single-, low-copy-, or unidentified TEs could be responsible for a subset of the CNV reported in this study. A more detailed annotation of barley TEs would be useful to understand the potential contribution of low-copy TEs to CNV in barley.

### CNV has the potential to contribute to phenotypic variation in barley

Our survey of barley CNV found that there are many examples of genes that are affected by structural variation. We detected 1,585 HC genes affected by CNV, and these often include UpCNV. This is consistent with previous observations of deletions being biased away from genes [4,57]. Stress and disease resistance genes, including many *NBS-LRR *genes, are over-represented in the 1,585 annotated genes. In agreement with previous studies [22,24], we found CNVs overlapping *R *genes to be clustered in the genome. Regions with the highest concentration of *R*-gene variants were located near the end of 1H and 7H short arms, which coincide with previously reported clusters of disease-resistance genes to multiple pathogens [58,59]. The short arm of 1H has not only been associated with leaf rust (*Rph4 *locus; [60]) and scald resistance (*Rrs14 *locus; [61]), but it also contains the well-known powdery mildew resistance complex locus *Mla*, which spans a region of at least 32 predicted genes, many of which are associated with plant defense responses [62]. The distal region of 7HS, also contains a high concentration of genes for resistance to stem rust (*Rpg1*; [63]), leaf stripe (*Rdg2a*; [64]), powdery mildew (*mlt*; [65]), and scald (*Rh2*; [66]).

Variation in gene copy numbers in barley has been previously reported for the boron transporter gene *Bot1 *[26] and the *CBF *genes clustered at the frost-tolerance locus *FR-2 *[36]. Although we could not test for possible variants at *Bot1*, as its sequence was not present on the array probes, we found evidence to support CNV at *CBF3 *[67], which may contribute to cold-tolerance in winter barley genotypes 'Igri' and 'Franka'. The knowledge of genes affected by CNV may contribute to our understanding of the molecular mechanisms for adaptation to biotic and abiotic stress in barley.

## Materials and methods

### Array design

A custom CGH array was designed by Roche NimbleGen (Roche NimbleGen, Inc., Madison, WI, USA) using 2.2 M contigs from a whole genome shotgun (WGS) assembly of barley cv. Morex (Assembly1, EMBL-EBI accession no. PRJNA30763). This was a first *de novo *assembly from cv. Morex using Illumina reads at 28× genome coverage. Variable length probes (56 - to 100-mers) were generated at a 10 bp step across the entire sequence space. Individual probes were repeat-masked by removing probes, which had an average 15-mer frequency >25, using a 15-mer frequency table generated from an initial assembly of the Morex genome. The repeat-masked probe set was compared back to the Morex genome assembly with SSAHA [68], using a minimum match size of 30 and allowing up to 5 indels/gap. Probe sequences with more than a single match in the genome were eliminated from further consideration. From the remaining probes, only sets of 10 non-repetitive and unique probes that were clustered in 200 bp regions throughout the sequence space (called 'contig fragments') were included in the array design. The final probe set contained a total of 2,116,690 probes representing 211,669 regions on 115,003 of the input Assembly1 contigs. Each region was separated by at least 500 bp from adjoining regions.

Two array designs were produced for the same set of probes, '101206_Barley_NS_CGH_HX1' and '110808_Barley_NS_CGH_HX1', the latter placing probes at different coordinates and it was used to validate results from the first design.

### Prediction of chromosomal positions

The 211,669 contig fragments from Morex WGS Assembly1 present on the array were aligned against publicly available WGS contigs integrated with the barley physical framework [38]. Alignment was done with MegaBLAST version 2.2.18 [69]. Only fragments with a unique high quality BLAST hit (HSP longer than 150 bp and identity >95%) were considered, which resulted in 203,240 contig fragments (96% of all fragments on the array) having a match to Assembly3 sequences. The remaining 4% of the contig fragments (8,429) had to be discarded due to missing or ambiguous alignments. For fragments with equivalents in the published WGS contigs [38], the anchoring information attached to their respective contigs in the barley physical framework was retrieved. This information included genetic and physical positions, chromosome arm assignments, and fingerprinting (FP) contigs. In this manner, 88.7% of the contig fragments could be assigned to a chromosome arm and 33.7% to an FP contig.

### Gene prediction and functional annotation

The intersection between contig fragments and annotated barley genes was determined. For this purpose, previously predicted genes [38] classified into high and low confidence were used. Protein sequences of high confidence genes were assigned functional annotations using the AFAWE pipeline [70]. Additionally, gene ontology (GO) terms for high confidence genes were computed with Interproscan version 5 beta [71]. Resulting general GO terms were converted into Plant GOslim categories using the Perl script map2slim [72]. GO term enrichment analyses were performed in agriGO [73,74] using all genes on the array as a reference.

### Array validation

DNA from the wheat cv. Chinese Spring (CS), barley (cv. Betzes), and a wheat-barley chromosome addition line which carries the 3HL chromosome arm of Betzes in the Chinese Spring background (CS-3HL), were isolated from leaf tissue and sent to the NimbleGen's Service Laboratory (Reykjavik, Iceland) for DNA labeling and array hybridization. To test the specificity and sensitivity of the designed array, equal amounts of CS-3HL and Betzes were labeled either with Cy3 or Cy5 and hybridized to two arrays (dye-swap replication) following NimbleGen's standard protocol [75]. Another two arrays (dye-swap technical replication) were hybridized with equal amounts of CS and Betzes as an experimental control. For both CS-3HL/Betzes and CS/Betzes contrasts, spatially corrected and normalized log2 ratios were obtained from each probe using the segMNT algorithm implemented in NimbleScan software v.2.6 (Roche NimbleGen, Inc., Madison, WI, USA). Probe log2 ratios were averaged by array contig fragment and then by contrast, and were displayed by barley chromosome/chromosome arms.

### Plant materials

Fourteen accessions were selected for this study. Eight cultivars from different geographic origins, growth habits, and end uses including: Barke and Betzes, which are European, 2-rowed, spring-type malting barleys; Harrington, a North American, 2-rowed, spring-type malting cultivar; Haruna Nijo, a Japanese, 2-rowed, spring-type malting barley; Bowman, a North American, 2-rowed spring-type feed barley; Igri, a European, 2-rowed winter-type malting cultivar; Steptoe, a North American, 6-rowed, spring-type feed barley; and Franka, a European, 6-rowed winter-type malting barley. The remaining genotypes comprised a geographical selection of six wild barley (*H. vulgare *ssp. *spontaneum*) accessions. All the information describing these 14 accessions can be found in Additional file 2, Table S2.

### DNA labeling and array hybridizations

DNAs from eight barley cultivars (Barke, Betzes, Harrington, Haruna Nijo, Bowman, Igri, Steptoe, and Franka), six wild barley accessions (Hsp11, Hsp248, Hsp278, Hsp357, Hsp462, and Hsp730), and the reference genotype 'Morex' were isolated from leaf tissue [76] and were labeled (Cy3 for sample; Cy5 for reference) and hybridized following the standard protocol provided by Roche NimbleGen [75]. Arrays were scanned immediately after washing at 2 μm resolution on the MS 200 Microarray Scanner and images were processed using Roche NimbleScan software v. 2.6 (Roche NimbleGen, Inc, Madison, WI, USA). Experimental Metrics Reports were generated from each of the images to assess the quality of our array experiments. Only images that met the suggested range of values for each of the parameters evaluated were considered for further analysis. Pair reports containing the raw signal intensities for each probe on the array were produced for each array, one for the Cy3 and one for the Cy5 images. The raw data were deposited in NCBI GEO under accession number GSE44293.

### Data normalization and linear modeling

Pair files exported from NimbleScan were imported into the Bioconductor statistical environment [77]. Array hybridization values were normalized to correct for inter-array and intra-array signal variations using Variance stabilization and calibration for microarray data (vsn, [78]). As both array platforms were designed using Morex as a reference, all individual replicated samples were exported as log2 (sample/reference) values. Normalized probe values were averaged across replicated samples and also across contig fragments for downstream analysis.

### Copy number analysis

The expectation maximization (EM) algorithm [79] was used to estimate the mixing proportion, mean, and variance associated with two predicted subdistributions found within the tested genotype *vs*. Morex fragments. For each contig fragment, the posterior probability that it occurred in each of the two distributions was determined. A stringent criterion was applied to identify CNVs: only contig fragments with a *P *>0.95 of falling into the first subdistribution and an absolute log2 ratio (sample/reference) >0.9 were considered significant. When the log2 ratio was positive, the variant was defined as 'UpCNV', while it was classified as 'DownCNV/PAV' when the ratio was negative.

### Validation of CNVs

A new array design ('110808_Barley_NS_CGH_HX1'), which had the same probes placed at different coordinates, was developed to validate CNVs identified in this study. Fifteen arrays produced high-quality data from genotypes Barke, Betzes, Bowman, Haruna Nijo, Steptoe, Hsp11, and Hsp730, and were used for validation. Data normalization, linear modeling, and analysis of CNV were done as explained above for the main array design. Percentages of CNVs validated were calculated.

A total of 26 DownCNV/PAVs and 17 UpCNVs were selected for PCR validation and primers were designed using BatchPrimer3 [80]. Validation of DownCNV/PAVs was conducted by semi-quantitative PCR using standardized and uniform PCR conditions, and amplicons were resolved on 2% agarose gels and visualized by ethidium bromide staining. UpCNVs were analyzed via quantitative PCR (qPCR) on an Applied Biosystems PRISM qPCR system utilizing the SYBR Green PCR Master Mix (Applied Biosystems). The relative copy number was determined by calculating the 2^-ΔΔCt ^values using data of three technical replicates. Contig fragment 'Contig_87926:7401-7601', encoding a pyruvate kinase, was used as internal control to normalize the data, and the fold-change values were referred to Morex. Primer pairs and PCR conditions for all 43 CNVs and the controls can be found in Additional file 2, Table S5).

### Identification of orthologous sequences from different barley cultivars

Comparison of DNA sequences containing CNVs between genotypes Morex (Assembly3, EMBL-EBI accession IDs, and CAJW010000001-CAJW012670738) and Barke (EMBL/ENA accession IDs CAJV010000001-CAJV012742077) was automated with a series of original Perl programs. The programs performed the following steps: as a reference, we used the Morex WGS contigs from which the array probes were derived. Those contigs were used in Blastn searches against Illumina sequence assemblies from WGS data of the barley cultivar Barke. The top Blastn hits were assumed to be the orthologous sequences as long as the sequence identity was >95% (this high stringency was chosen to avoid non-specific hits caused by repeats). In cases where the Morex contig was longer than the orthologous Barke contigs, the Barke sequences were concatenated into supercontigs to cover as much of the Morex reference sequence as possible. The Morex and Barke sequences were then aligned with the program Water [81], which is an implementation of the Smith-Waterman algorithm. From this sequence alignment, the contig fragment regions targeted by the probes were extracted and evaluated.

For the analysis, we used only contigs which contained multiple contig fragments targeted by the CGH array. Furthermore, we required that at least one contig fragment affected by a CNV was flanked by contig fragments not affected by CNV. This was done to select contigs that contain the entire CNV flanked by non-variable sequences.

### Data access

WGS Assembly1 of barley cv. Morex was deposited at EMBL-EBI, under accession PRJNA30763. The assembly of cultivar Barke and Assembly3 of cultivar Morex have been published before and are available under EMBL/ENA accession IDs CAJV010000001-CAJV012742077 and CAJW010000001-CAJW012670738, respectively. Both assemblies can also be downloaded from Helmholtz Zentrum München [82]. Design files of the barley CGH custom array '101206_Barley_NS_CGH_HX1' and raw. pair files resulted from array hybridizations have been submitted to NCBI GEO under accession GSE44293 [83].

## Abbreviations

CBF: C-repeat binding factor; CGH: comparative genomic hybridization; CNV: copy number variation; DSB: double-strand break; FoSTeS: fork stalling and template switching; MMBIR: microhomology-mediated break-induced replication; MMEJ: microhomology-mediated end-joining; NAHR: non-allelic homologous recombination; NHEJ: non-homologous end-joining; PAV: presence/absence variation; RIL: recombinant inbred line; SDSA: synthesis-dependent strand annealing; SNH: segregation of non-allelic homologous; SNP: single-nucleotide polymorphism; SNV: single-nucleotide variation; SSA: single-stranded annealing; TE: transposable element; WGS: whole-genome shotgun.

## Competing interests

The authors from Roche NimbleGen Inc (TAR and JAJ) recognize a competing interest in this publication as employees of the company.

## Authors' contributions

MMA performed the CGH experiments. NS, TAR, GJM, and JAJ conceived the experiments. TAR and JAJ designed the custom CGH array and performed the pilot experiments. MMA and SRE analyzed array CGH data. ST and MP generated WGS of barley. TW performed sequence analyses of CNVs. MM performed Assembly1-Assembly3 alignments. MM, BS, and US performed computational analyses. RA, TN, and KFXM provided physical map anchoring info of the CGH data. MS performed GO annotation of CGH targets. NMS, SRE, GJM, and NS helped with the interpretation of the data. MMA wrote the paper with helpful contributions from NS, GJM, NMS, MM, and TW. All authors read and approved the final manuscript.

## Supplementary Material

Additional file 1 **PDF file containing all supplementary figures and their legends**. Click here for file

Additional file 2 **Excel file containing all supplementary tables and their legends**. Click here for file
