# Supplementary material for: Distribution, functional impact, and origin mechanisms of copy number variation in the barley genome
Source: Genome Biol. 2013 Jun 12;14(6):R58. doi: 10.1186/gb-2013-14-6-r58 (PMC3706897; doi:10.1186/gb-2013-14-6-r58)
Supplement: Additional file 1 — PDF file containing all supplementary figures and their legends. [file gb-2013-14-6-r58-S1.PDF]

Figure S1. Barley CGH array design. The barley WGS contig_12361 (grey bar) is represented on the array by six 200 bp contig fragments (black bars). Ten non-repetitive and unique probes (black lines) representing each of the six contig fragments were included in the array design. The WGS contig contains a gene (MLOC_58402.1, in green), which encodes a zinc finger CCCH domain-containing protein 36 (high-confidence gene prediction). In this case, all contig fragments targeted by the array were contained in the gene sequence, and three of them in exons.

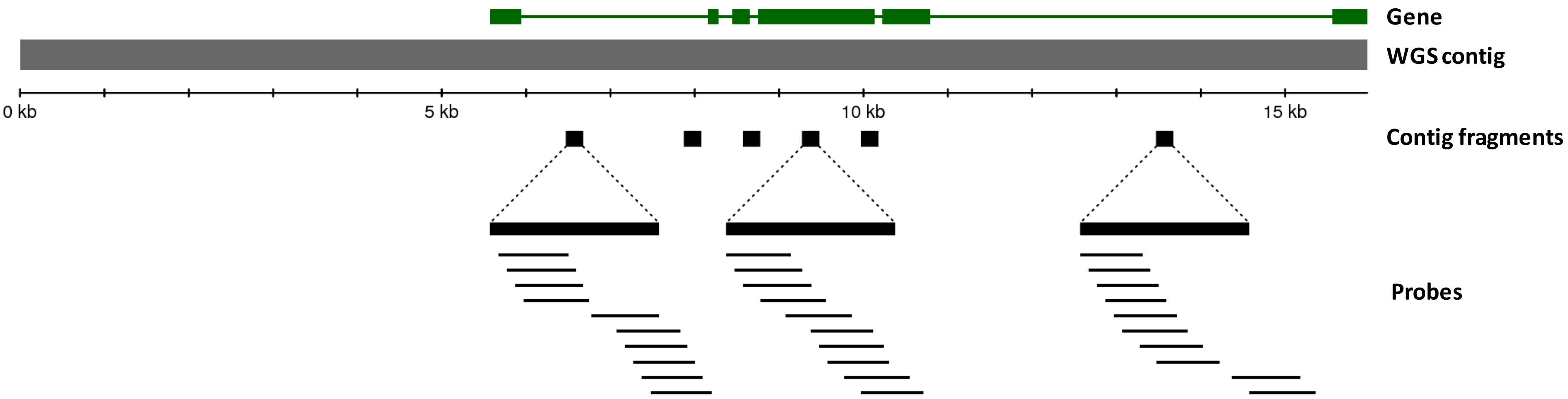

Figure S2. Distribution of contig fragments by barley chromosome / chromosome arms. The plot shows $\log_2$(3HL wheat-Betzes addition line /Betzes) (A) and $\log_2$(wheat/Betzes) (B) for all 211669 contig fragments on the array. 88.7% of the contig fragments are assigned to chromosome 1H and arms of chromosomes 2H-7H.

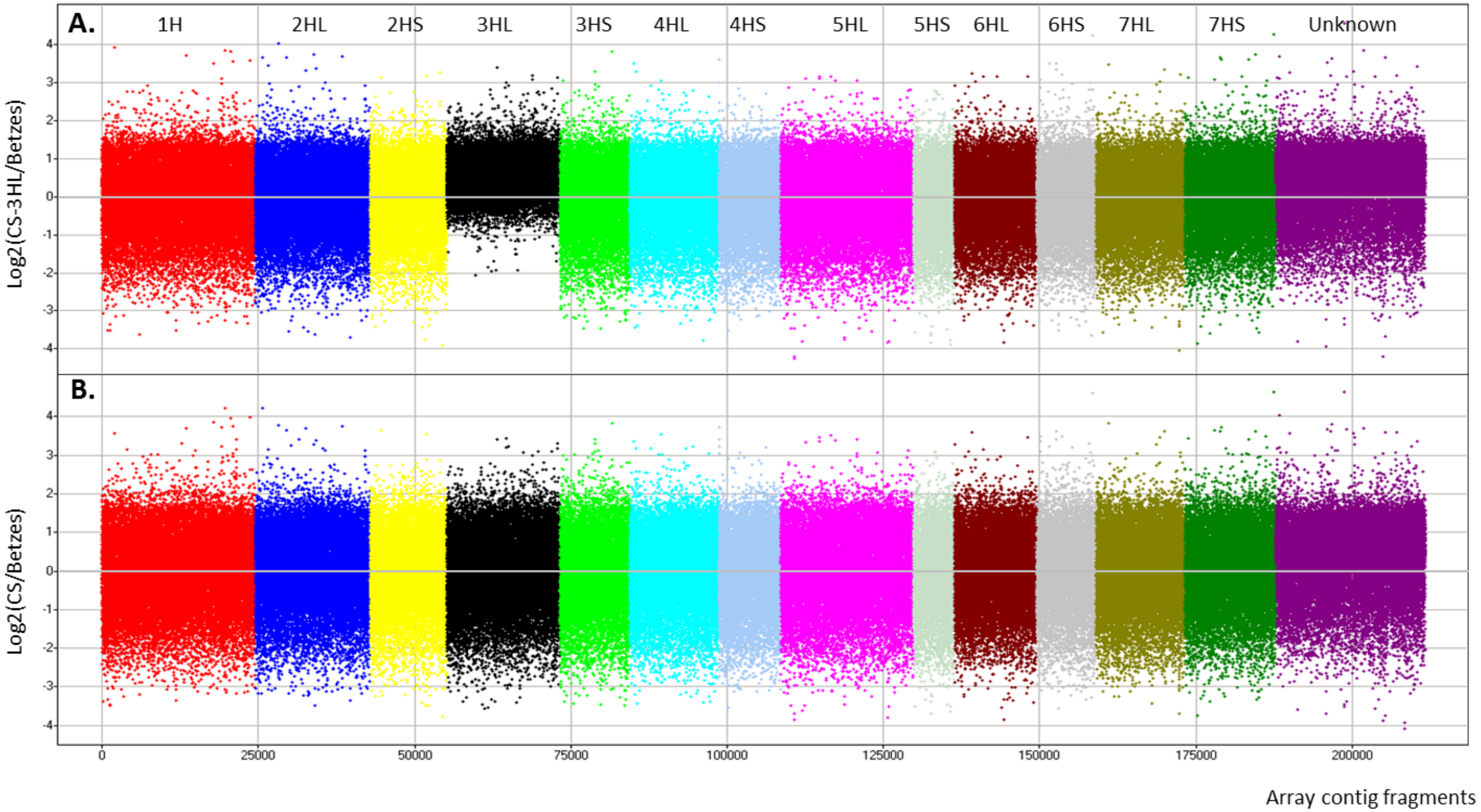

Figure S3. Unrooted neighbour-joining (NJ) tree of all genotypes used in the study based on SNP data. The NJ tree, based on Manhattan distances between individuals, was generated with the R package 'ape' (Paradis et al. 2004). SNP data was obtained by genotyping the accessions included in the study with the iSelect Illumina platform (Comadran et al. 2012), which generated high-quality genotype calls for 6,938 SNPs.
Genotypes are clustered into two main groups of subspecies accessions. Cultivar 'Morex' (reference) and 'Steptoe' (6-row barleys), were positioned closer to the wild barleys than the rest of the cultivars, followed by the winter-type cultivars 'Franka' and 'Igri'. The rest of the cultivars are 2-row spring types. The subclustering of wild barleys is mostly determined by geographical origin. The observed higher phylogenetic resolution in the cluster of cultivars is due to the expected ascertainment bias caused by the use of barley cultivars as discovery panels in the SNP platform that we used (Moragues et al. 2010).

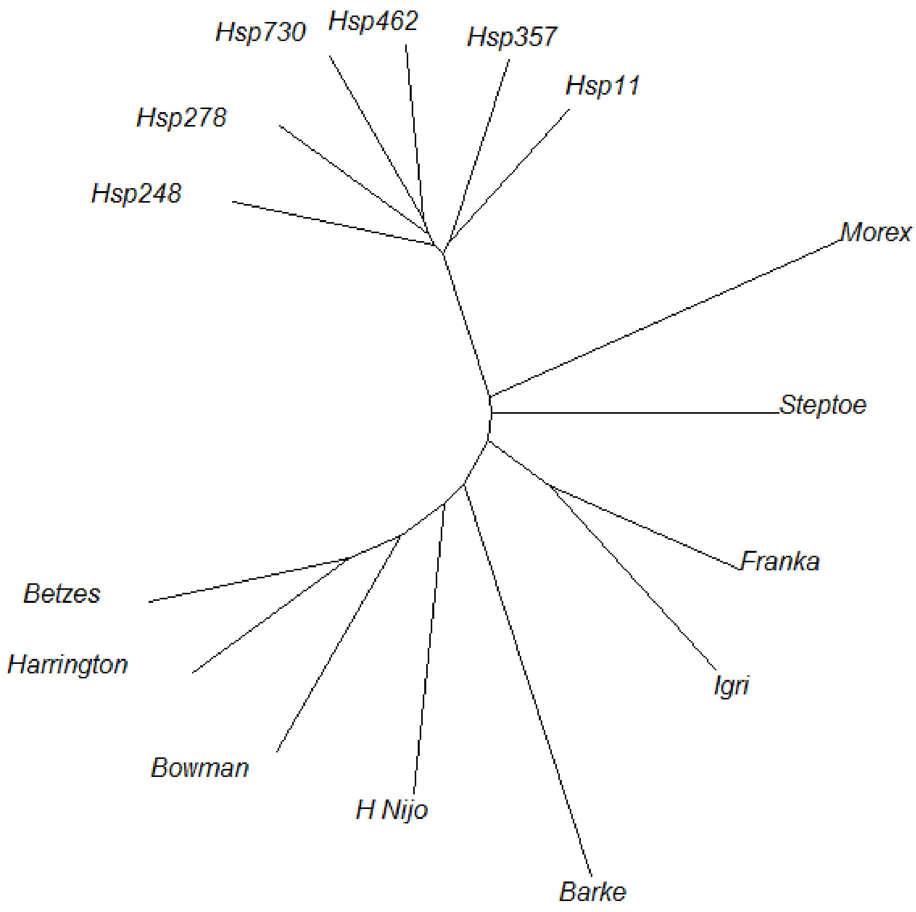

Figure S4: Distribution of CNV/PAV by chromosome for all fourteen genotypes used in the study. The bars represent percentages of CNVs assigned to each chromosome relative to the total number of contig fragments present on the corresponding chromosome.

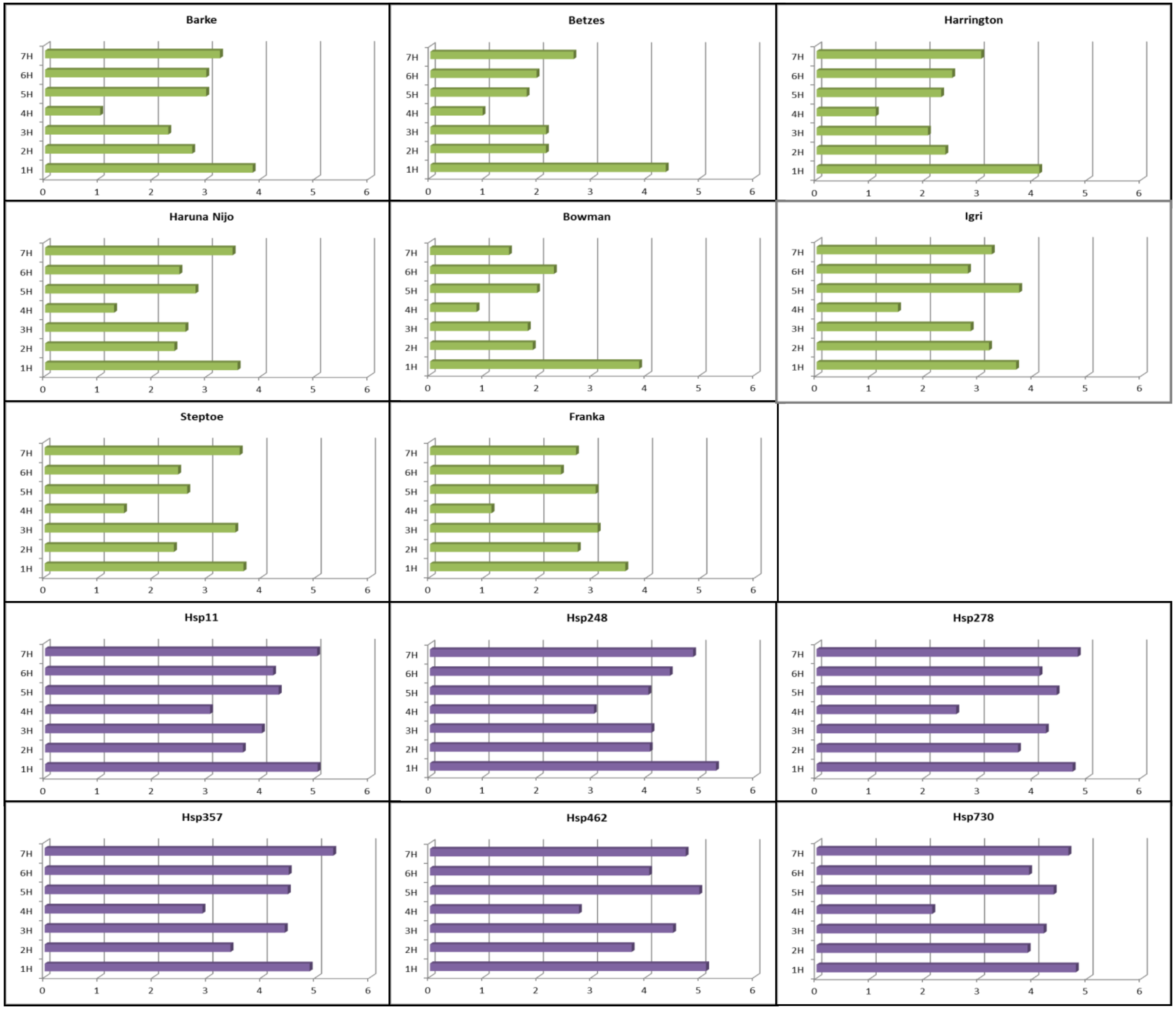

Figure S5: Visualization of CNV throughout barley chromosome 7H. The distribution of CNVs in the fourteen accessions used for the study is shown, with cultivars on the left and wild barleys on the right. Each data point represents the log2 ratio (genotype/Morex) of a targeted 200 bp contig fragment. UpCNVs are colored in blue, while DownCNV/PAVs are shown in red. The black arrow indicates a chromosome region with complete absence of CNV.

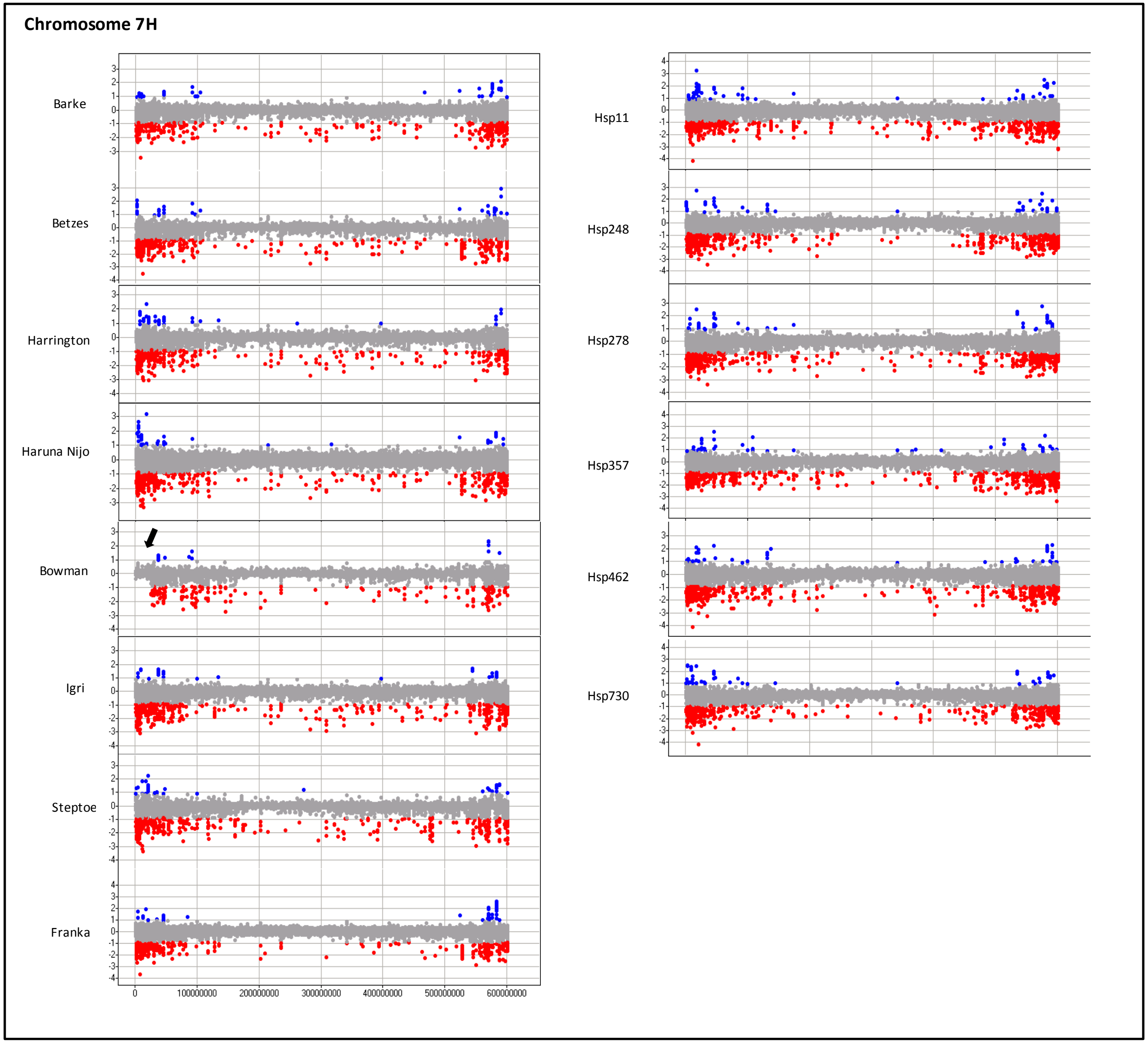

Figure S6: Example of four DownCNV/PAVs covering a 4.4 kb region of the barley chromosome 2H. Variants are located between 700 and 5100 bp of the WGS contig_42392. The WGS contig is represented by another four contig fragments that do not show CNV. Log2 ratios of the 200 bp contig fragments are shown, with green representing cultivar Harrington, which contains the CNVs, and blue representing genotype Hsp730, as an example of genotype not containing these structural variants.

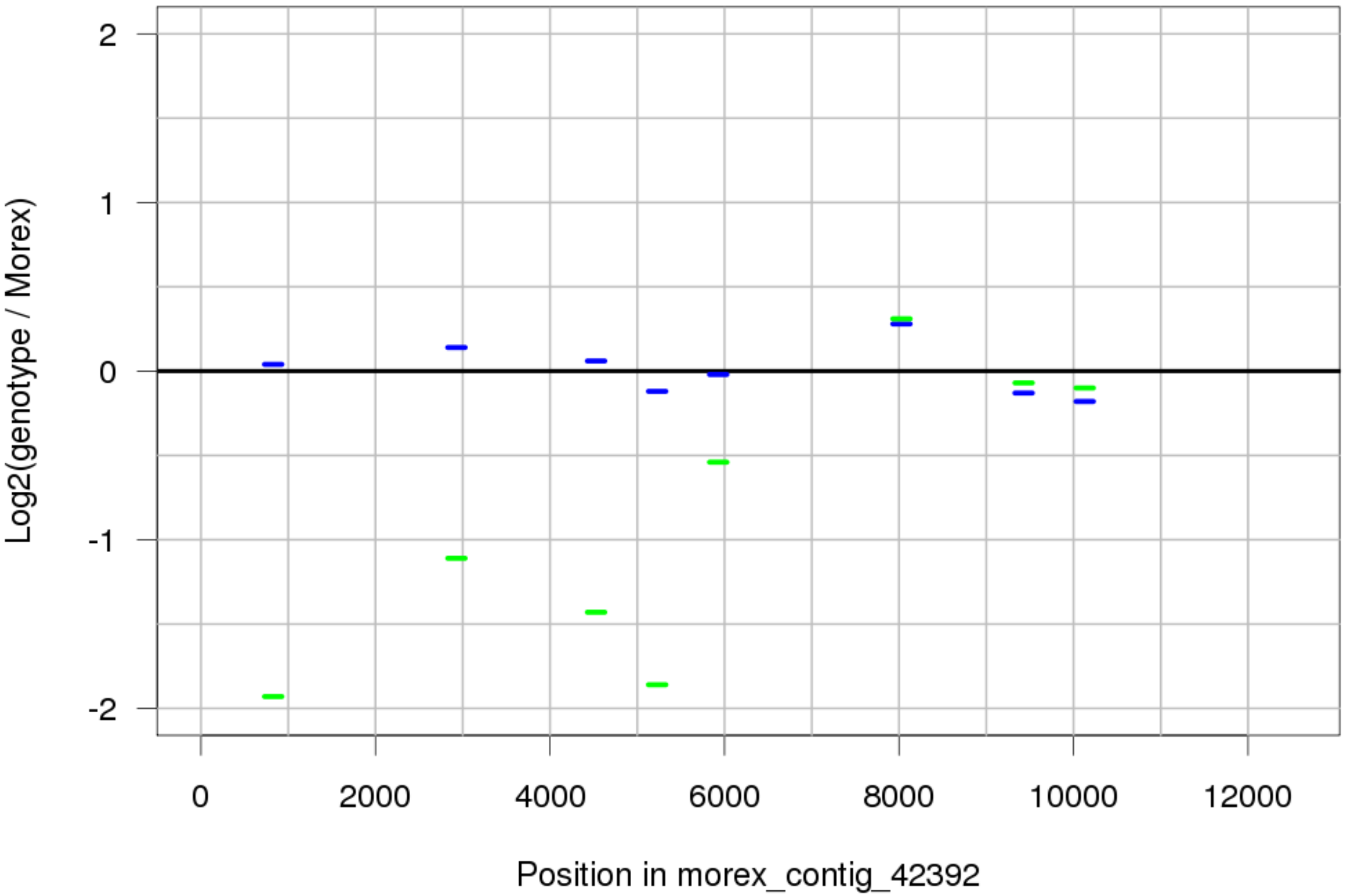

Figure S7: Examples of DownCNV/PAVs and UpCNVs validated by semi-quantitative and quantitative PCR. (A) DownCNV/PAVs validated by semi-quantitative PCR. The upper plots show agarose gel images of two amplified contig fragments, while lower plots represent log2 ratios obtained from the CGH experiment. (B) UpCNVs validated by qPCR. Blue boxes represent CGH data, while the red dots show qPCR results. A log2 of $2^{-\Delta\Delta Ct}$ qPCR values was calculated to match the scales.

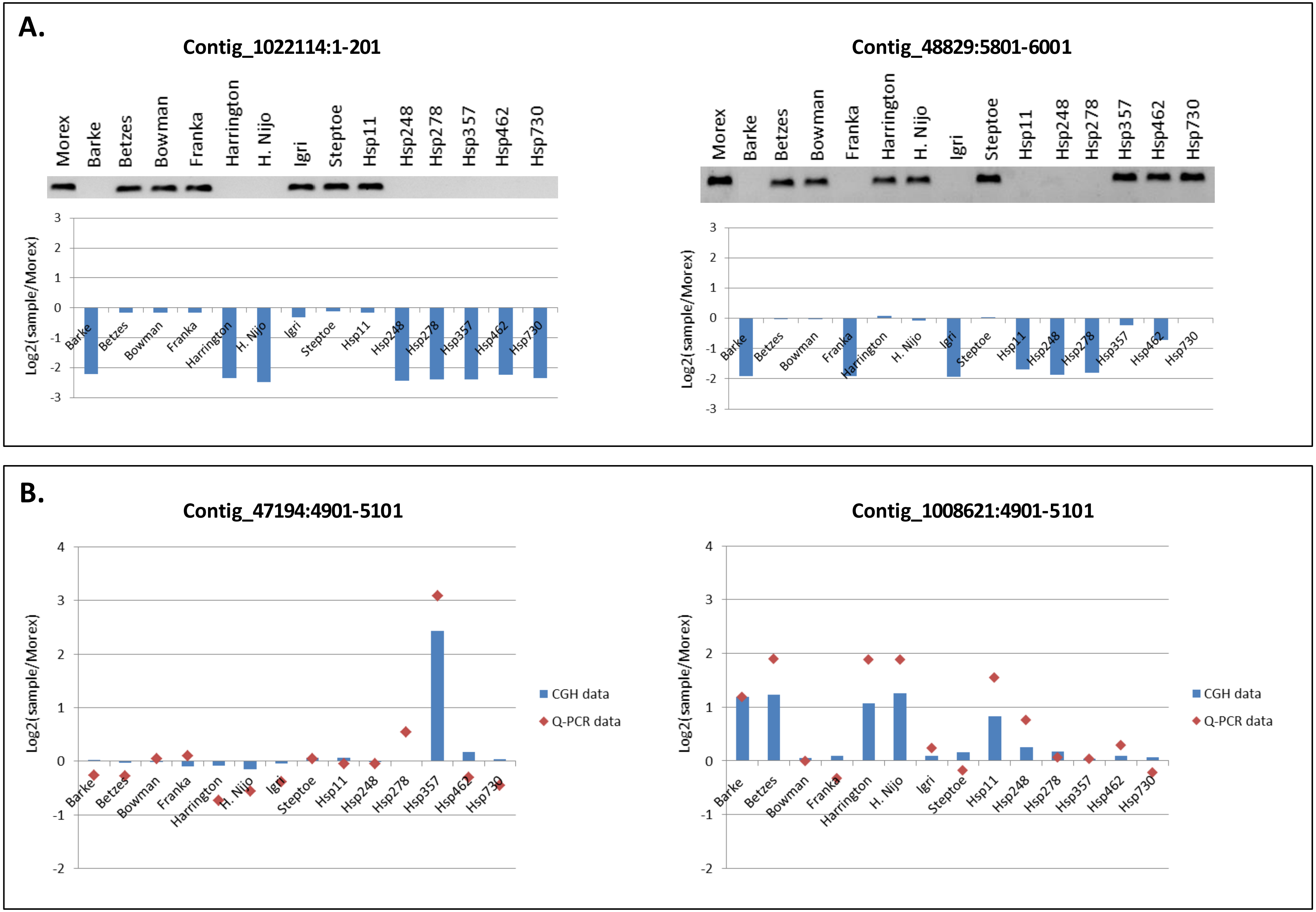

Figure S8: **Chromosome location and number of genotypes containing CNVs in *R*-genes.**

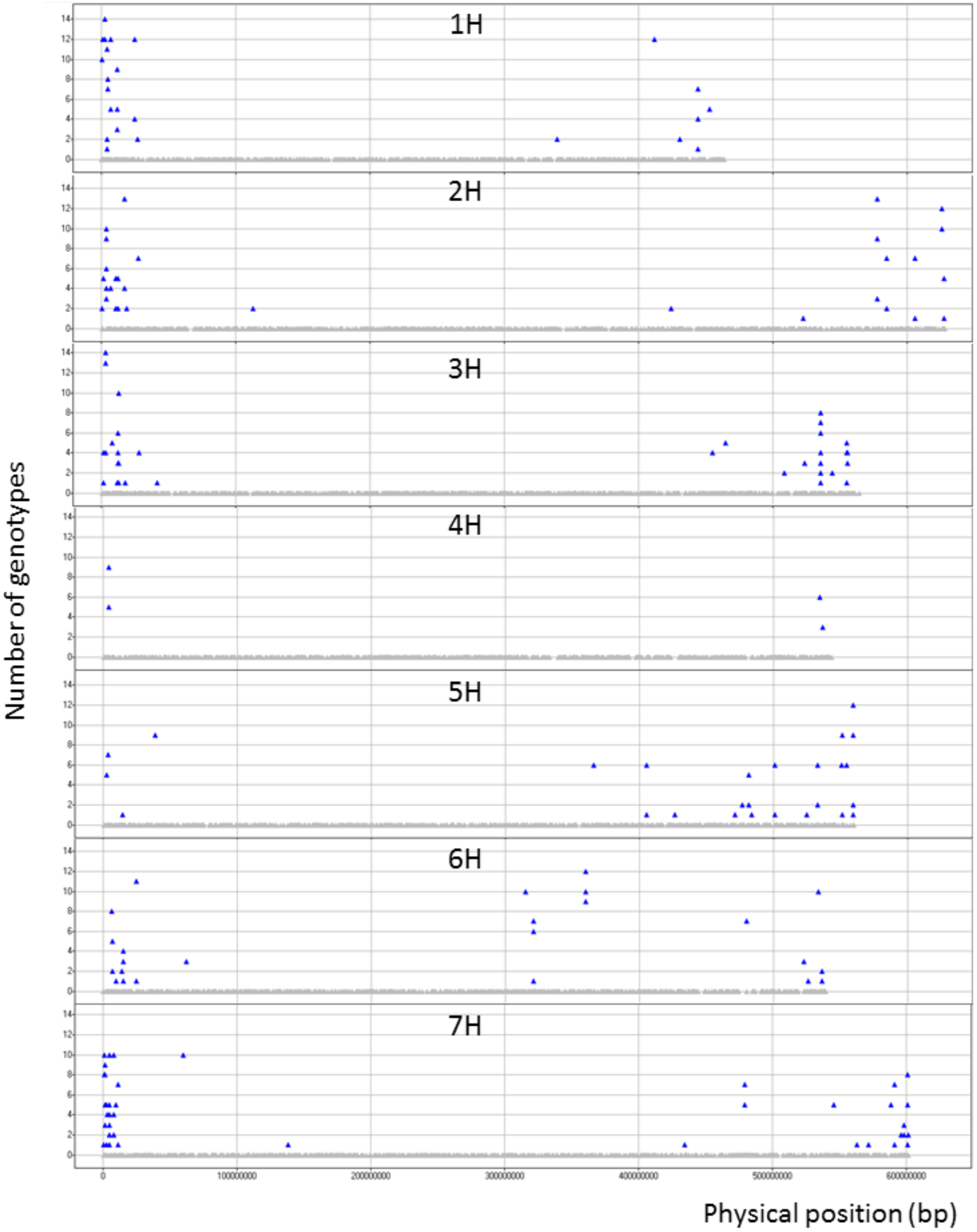

Figure S9: Difference between the number of CNVs present in wild and cultivated barley for each chromosome. The line deviates from zero where there is a difference between the two subspecies, with positive values indicating higher numbers of variants in wild barley and negative ones indicating regions where there are more CNVs in cultivated barley. Numbers were calculated for 1.5Mbp windows.

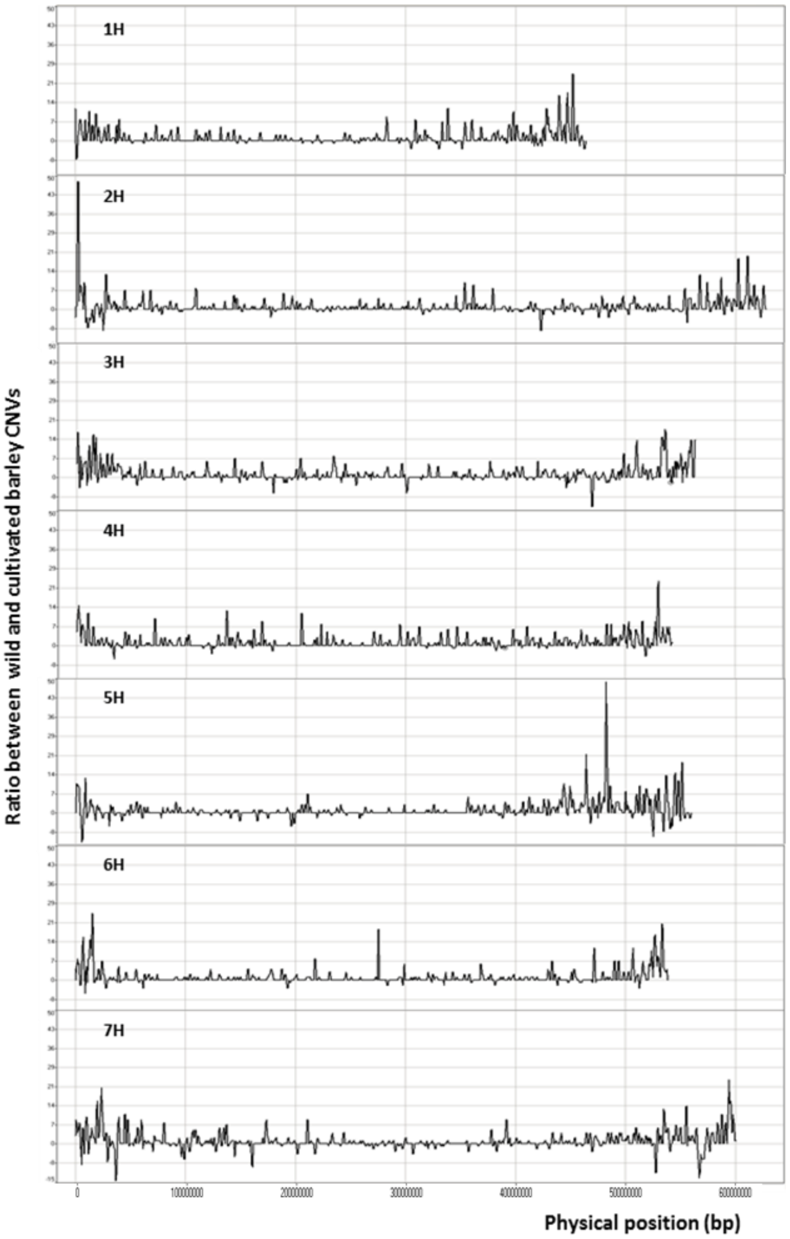

References

- Comadran J, Kilian B, Russell J, Ramsay L, Stein N, Ganal M, Shaw P, Bayer M, Thomas W, Marshall D, Hedley P, Tondelli A, Pecchioni N, Francia E, Korzun V, Walther A, Waugh R: **Natural variation in a homolog of Antirrhinum CENTRORADIALIS contributed to spring growth habit and environmental adaptation in cultivated barley.** *Nat Genet* 2012, **44:** 1388-1392.

- Moragues M, Comadran J, Waugh R, Milne I, Flavell AJ, Russell JR: **Effects of ascertainment bias and marker number on estimations of barley diversity from high-throughput SNP genotype data.** *Theor Appl Genet* 2010, **120:** 1525-1534.

- Paradis E, Claude J, Strimmer K: **APE: analyses of phylogenetics and evolution in R language.** *Bioinformatics* 2004, **20**: 289-290.
