# Supplementary material for: Distribution, functional impact, and origin mechanisms of copy number variation in the barley genome
Source: Genome Biol. 2013 Jun 12;14(6):R58. doi: 10.1186/gb-2013-14-6-r58 (PMC3706897; doi:10.1186/gb-2013-14-6-r58)
Supplement: Additional file 2 — Excel file containing all supplementary tables and their legends. [file gb-2013-14-6-r58-S2.DOCX]

Link to Additional file 2: <ftp://ftp.ipk-gatersleben.de/arraydesign/Additional%20file%202.xlsx>
